# Supplementary material for: Differential plasma exosomal long non-coding RNAs expression profiles and their emerging role in E-cigarette users, cigarette, waterpipe, and dual smokers
Source: PLoS One. 2020 Dec 8;15(12):e0243065. doi: 10.1371/journal.pone.0243065 (PMC7723270; doi:10.1371/journal.pone.0243065)
Supplement: S1 Fig — The top 6 enriched: (A) Biological pathway and (B) Site of expression for the significant lncRNAs and possible gene targets on pairwise comparisons between (i) E-cigarette users vs. Non-smokers, (ii) Waterpipe smokers vs. Non-smokers, and (iii) Dual smoker vs Non-smokers. NS, non-smoker; CS, cigarette smoker; E-cig, Electronic cigarette users; WP, waterpipe smoker; CSWP: Dual Smoker. (PDF) [file pone.0243065.s001.pdf]

Supplementary Figures

Differential plasma exosomal long non-coding RNAs expression profiles and their emerging role in E-cig users, cigarette, waterpipe, and dual smokers

Gagandeep Kaur, Kameshwar Singh, Krishna P. Maremanda,
Dongmei Li, Hitendra S. Chand and Irfan Rahman

Figure S1 A-B (i)

A. Biological Pathways

B. Site of Expression

(i) E-cig vs NS

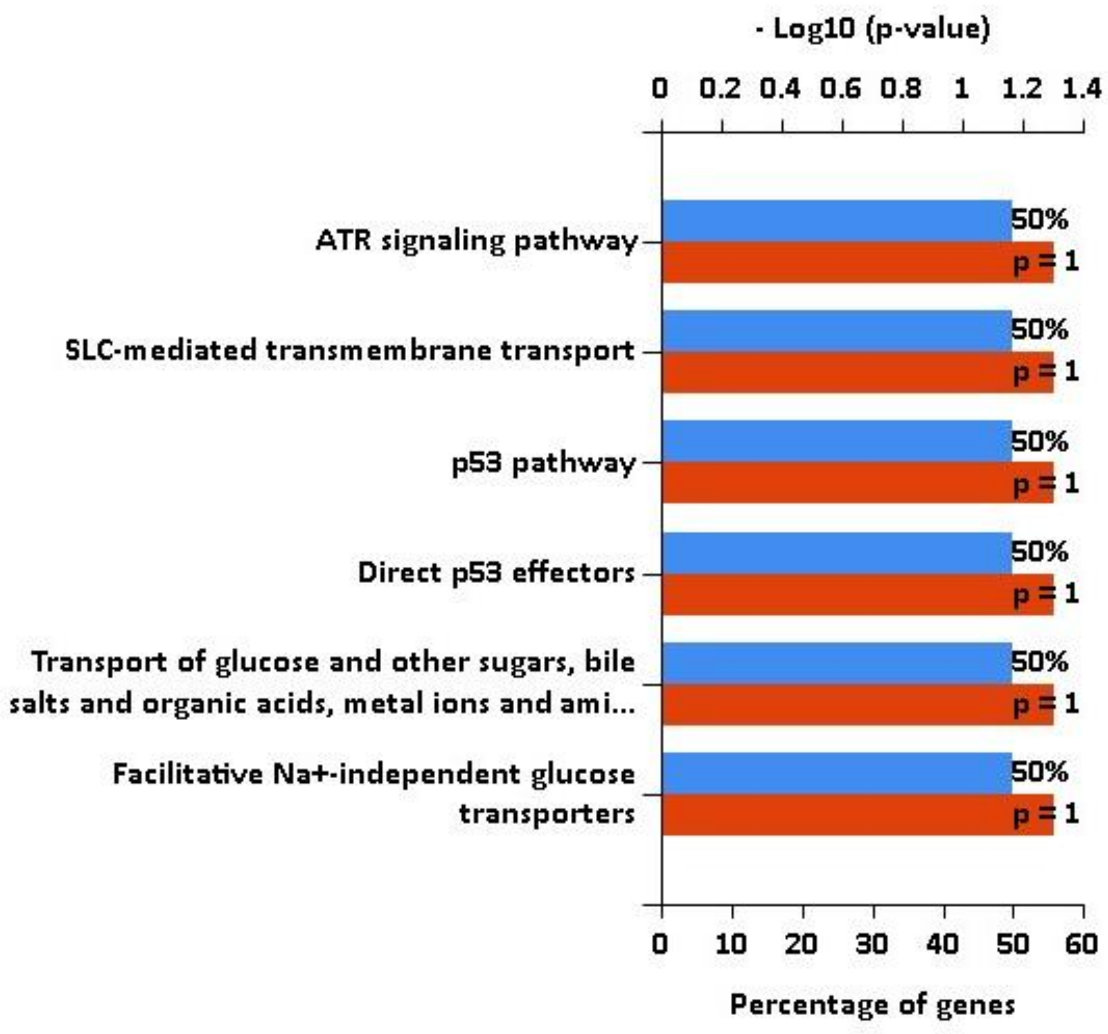

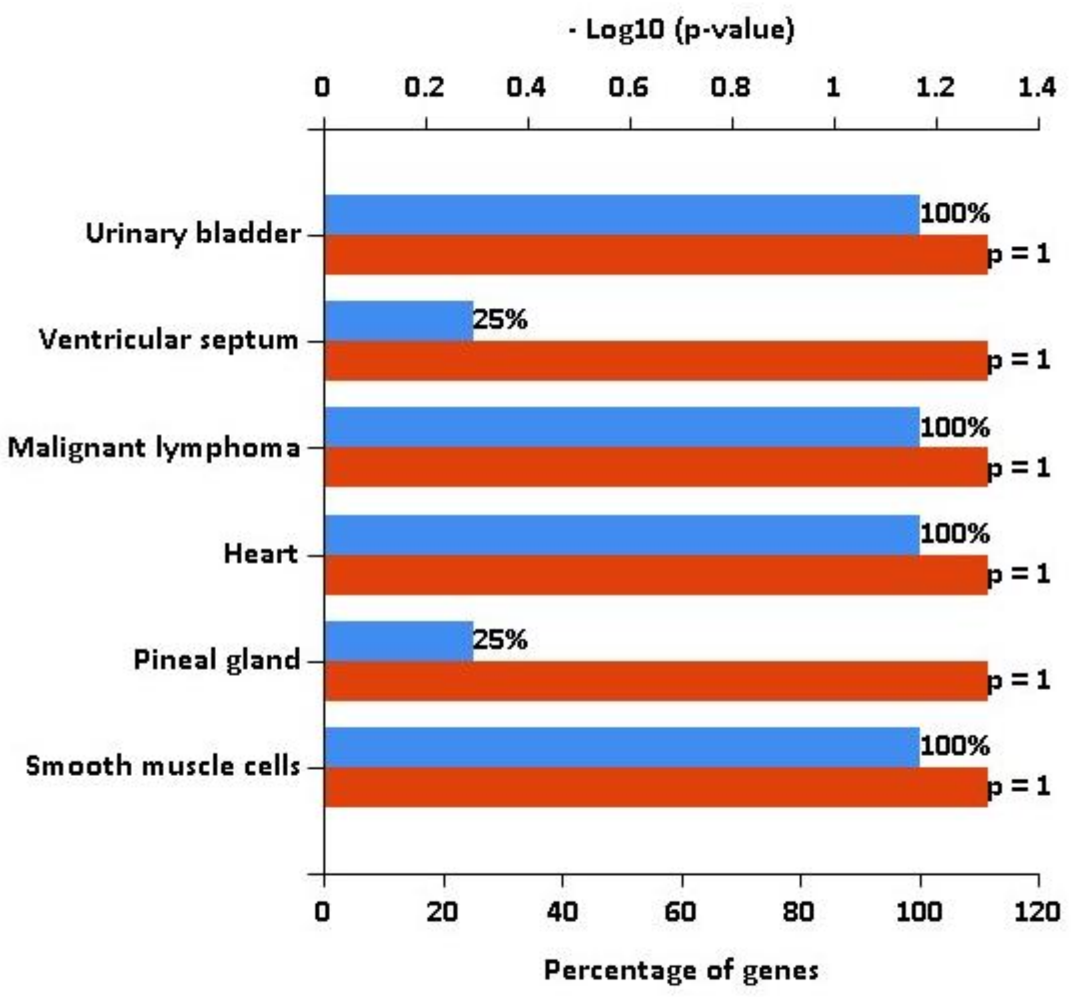

Percentage of genes
p-Value
p-Value <= 0.05 of the reference

Figure S1 A-B (ii)

A. Biological Pathways

B. Site of Expression

(ii) WP vs NS

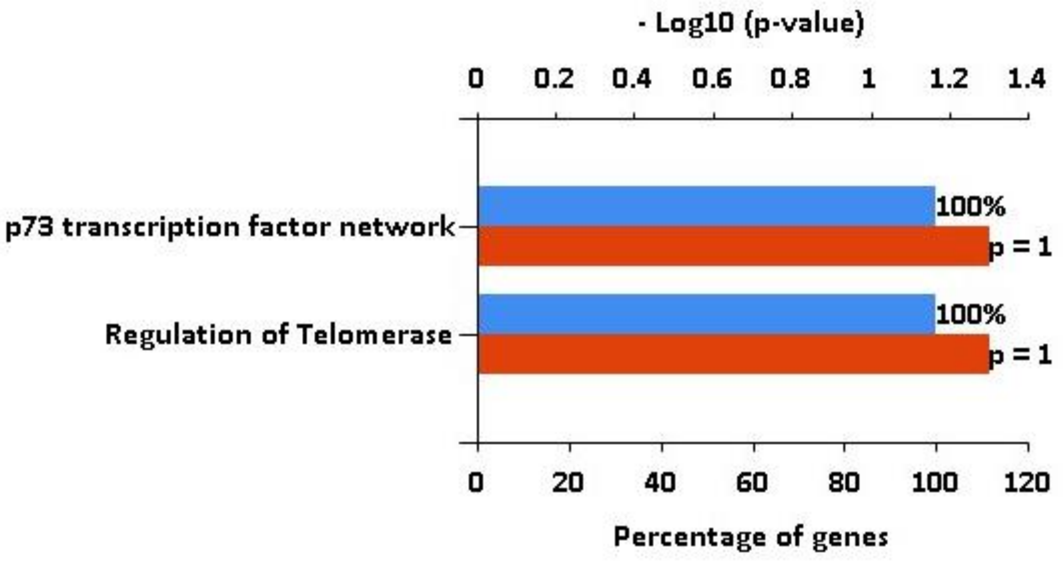

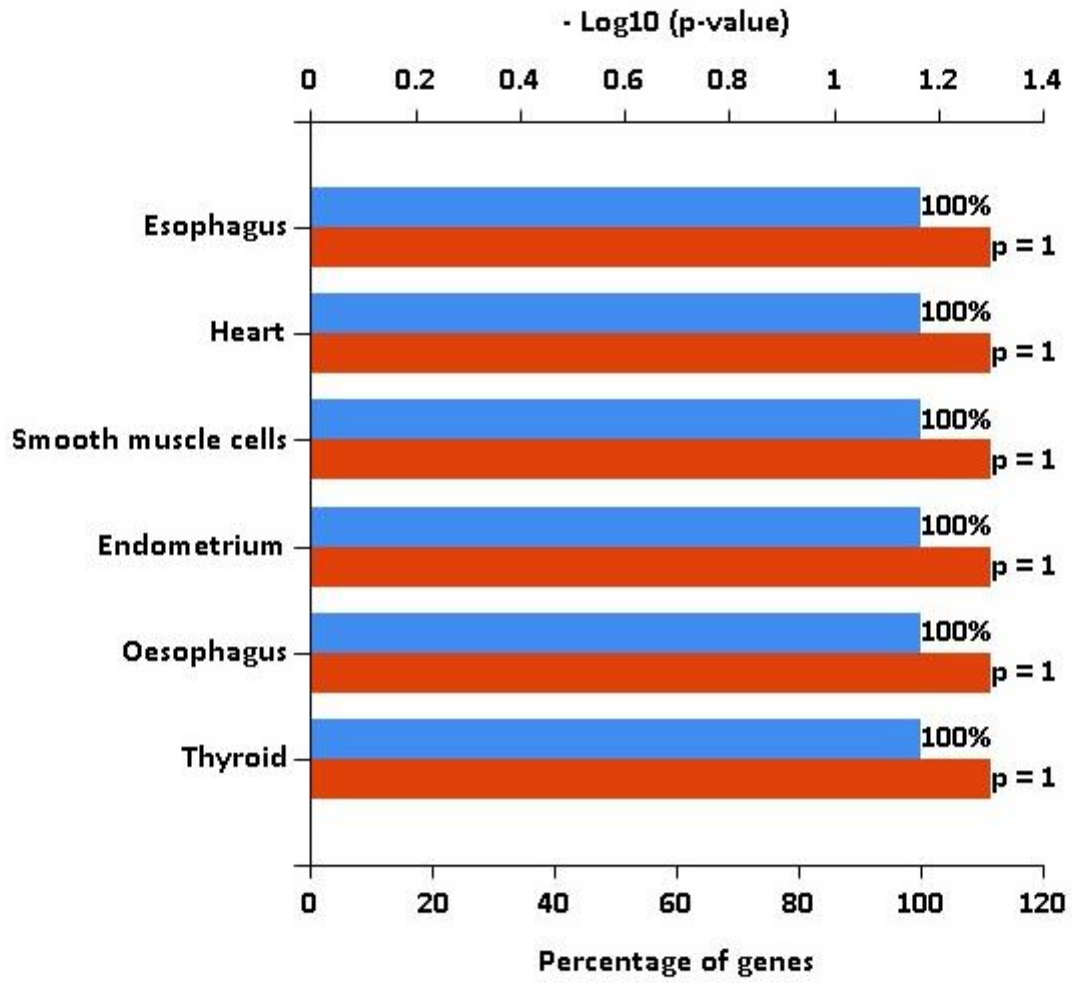

Percentage of genes
p-Value
p-Value <= 0.05 of the reference

Figure S1 A-B (iii)

A. Biological Pathways

B. Site of Expression

(iii) CSWP vs NS

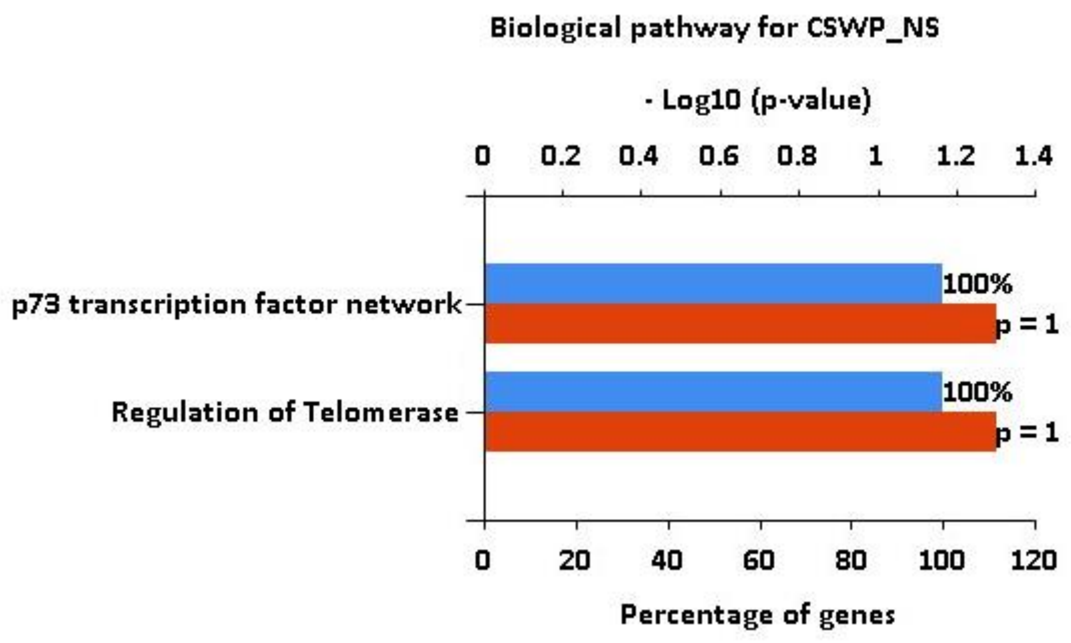

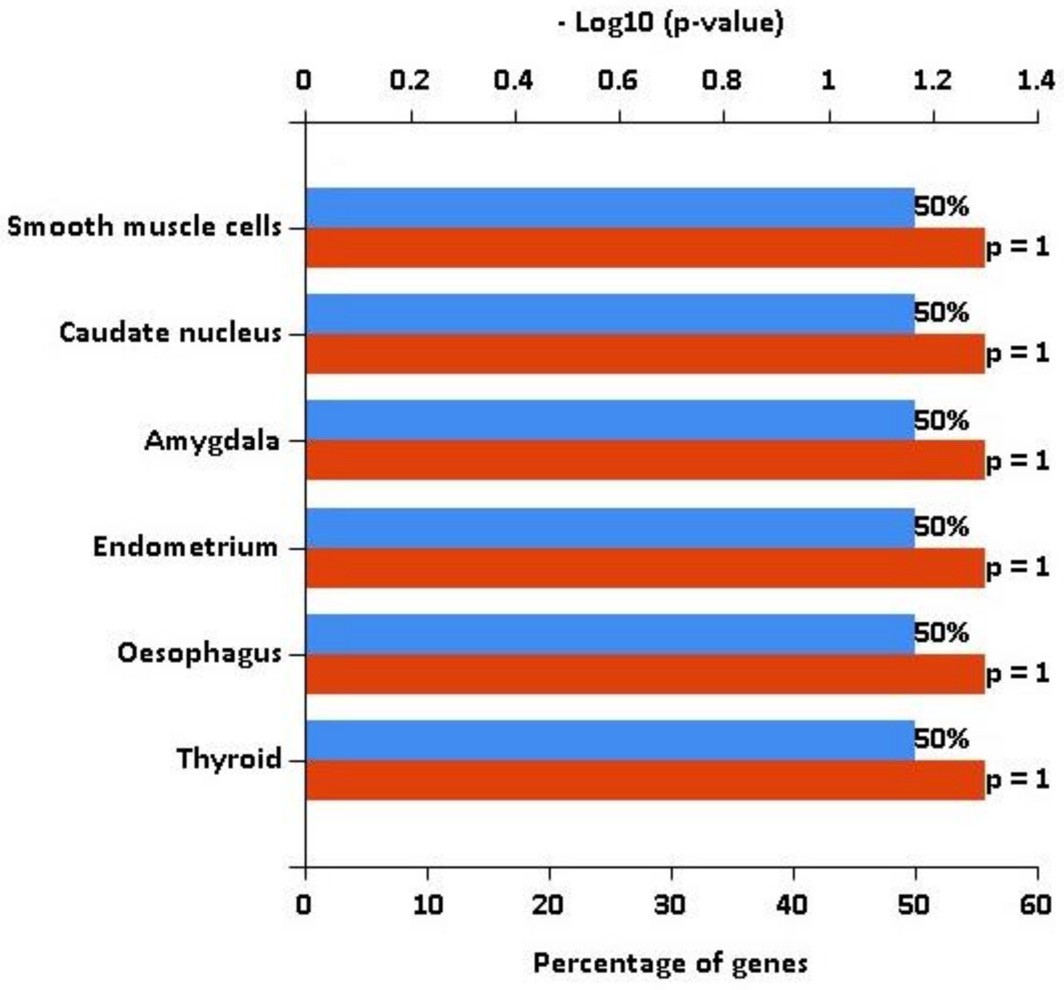

Percentage of genes
p-Value
p-Value <= 0.05 of the reference
